# Improved Feature Parameter Extraction from Speech Signals Using Machine Learning Algorithm

**DOI:** 10.3390/s22218122

**Published:** 2022-10-24

**Authors:** Akmalbek Bobomirzaevich Abdusalomov, Furkat Safarov, Mekhriddin Rakhimov, Boburkhon Turaev, Taeg Keun Whangbo

**Affiliations:** 1 Department of Computer Engineering, Gachon University, Sujeong-gu, Seongnam-si 461-701, Gyeonggi-do, Korea; 2 Department of Artificial Intelligence, Tashkent University of Information Technologies Named after Muhammad Al-Khwarizmi, Tashkent 100200, Uzbekistan

**Keywords:** speech recognition, parallel computing, distributed computing, multicore processor, feature extraction, spectral analysis

## Abstract

Speech recognition refers to the capability of software or hardware to receive a speech signal, identify the speaker’s features in the speech signal, and recognize the speaker thereafter. In general, the speech recognition process involves three main steps: acoustic processing, feature extraction, and classification/recognition. The purpose of feature extraction is to illustrate a speech signal using a predetermined number of signal components. This is because all information in the acoustic signal is excessively cumbersome to handle, and some information is irrelevant in the identification task. This study proposes a machine learning-based approach that performs feature parameter extraction from speech signals to improve the performance of speech recognition applications in real-time smart city environments. Moreover, the principle of mapping a block of main memory to the cache is used efficiently to reduce computing time. The block size of cache memory is a parameter that strongly affects the cache performance. In particular, the implementation of such processes in real-time systems requires a high computation speed. Processing speed plays an important role in speech recognition in real-time systems. It requires the use of modern technologies and fast algorithms that increase the acceleration in extracting the feature parameters from speech signals. Problems with overclocking during the digital processing of speech signals have yet to be completely resolved. The experimental results demonstrate that the proposed method successfully extracts the signal features and achieves seamless classification performance compared to other conventional speech recognition algorithms.

## 1. Introduction

The establishment of communication approaches between humans and computer technology is a critical task in modern artificial intelligence. One of the easiest methods for users to enter information is through speech signals. Therefore, speech signal processing technology and its tools have become a necessary part of the information society. Speech recognition is an essential research aspect of speech signal processing and a vital human–computer interaction technique. Speech signals contain semantic, personal, and environmental information [1]. The general approach to speech signal processing is to use short-term analysis, in which the signal is divided into time windows of a fixed size, assuming that the signal parameters do not change. An overlap is placed between windows to obtain a more accurate signal representation. Feature extraction algorithms such as spectral analysis and linear prediction are applied to each window. However, these processes should also consider speed and time.

Timing is a significant concern in several areas of real-time signal processing. The processing speed ratio can be increased by efficiently using processor resources and developing new and fast algorithms to reduce the digital processing time. However, high-performance computing problems have not yet been entirely resolved [2]. Moreover, substantial progress has been made in automatic speech recognition with the development of digital signal processing hardware and software. However, despite these advances, machines cannot match the performance of their human counterparts in terms of accuracy and speed recognition, particularly in speaker-independent speech recognition. Thus, a large amount of research that has been conducted on speech recognition has focused on speech recognition problems that are independent of the speaker, owing to the wide range of applications and limitations of the available speech recognition techniques [3].

In summary, the main contributions of the study are as follows:

- The process of identifying a person by speech signals consists of several stages. Among them, there are steps that require more calculation time. This is spectral analysis. One of the parameters that is especially important in machine learning algorithms is the training time. In this work, we found that the process of extracting features is important for the identification of speech signals. Additionally, it takes a lot of time in machine learning. It is established that the process of spectral analysis consists of independent operations, and parallelization is possible.
- Experiments have shown that FFT and DCT spectral transformations give good results in spectral analysis. The spectral analysis process is valid for each segment of the speech signal. This allows us to clearly distinguish these features from the speech signal. With the help of the OpenMP and TBB tools, a parallel computing algorithm was developed that made it possible to speed up the calculations.
- The segment size is important when dividing a speech signal into segments. During the experiments, it was found that the size of the segment of the speech signal depends on the cache memory of the central processor. In particular, matching segment to cache size in multicore processors has been found to have a positive effect on computational speed in machine learning.
- A new parallel implementation method is proposed for spectral transformations of signals for feature parameter extraction from speech signals using a machine learning algorithm on multicore processors using TBB and OpenMP.
- It was also found that the effective use of the principle of mapping the main memory block to the cache and the size of the cache memory block can increase the processing speed.

The remainder of this paper is organized as follows. Section 2 reviews existing studies for extracting specific speech signal properties. Section 3 describes the feature extraction steps with experiments. Section 4 presents the proposed high-performance computational algorithm for extracting the feature parameters from speech signals in detail. The experimental results based on our implementation are discussed in Section 5. Section 6 highlights the limitations of the proposed method. Finally, Section 7 concludes the study by summarizing the findings and noting future research directions.

## 2. Related Works

Novel methods, algorithms, and more modern applications are currently being developed and improved for the segmentation of speech signals and the calculation of parametric indicators of selected fragments, thereby creating a spectrogram of speech signals using spectral analysis [4,5,6,7]. Speaker identification with diversified voice clips across the globe is a crucial and challenging task, especially in extracting vigorous and discriminative features [8]. A novel Mel Frequency Cepstral Coefficients (MFCC) feature extraction system that is quicker and more energy efficient than the traditional MFCC realization was proposed by Korkmaz et al. [9]. Instead of using a high-pass pre-emphasizing filter, they used a low-pass filter. They implemented a bandpass filter with a high-pass filter because pre-emphasizing is needed to boost the signal’s energy in high frequencies. In our previous work [10], we presented a novel MFCC-based method for speaker identification. Instead of using conventional MFCCs, we employ pixels from the Mel spectrogram as our feature set. The spectrogram was transformed into a 2-D array to create a new dataset. To identify the speaker, several learning algorithms were put into practice with a 10-fold cross-validation technique. With the help of a multi-layer perceptron (MLP) with a tanh activation function, the best accuracy of 90.2% was achieved.

Feature extraction is accomplished by changing the speech waveform to a form of parametric representation at a relatively lesser data rate for subsequent processing and analysis [11,12,13,14]. Feature extraction approaches usually yield a multidimensional feature vector for every speech signal. Feature extraction is the most relevant portion of speaker recognition. Features of speech have a vital part in the segregation of a speaker from others. Feature extraction reduces the magnitude of the speech signal, devoid of causing any damage to the power of the speech signal [15,16,17]. In [18], the authors introduced a new approach that exploits the fine-tuning of the size and shift parameters of the spectral analysis window used to compute the initial short-time Fourier transform to improve the performance of a speaker-dependent automatic speech recognition (ASR) system. In the absence of doctors, laypeople can employ decision assistance systems that were created using artificial intelligence approaches. Early and non-invasive heart status detection can be accomplished using phonocardiogram (PCG) signals [19,20,21].

Convolutional neural networks (CNNs) have been demonstrated to successfully simulate structural locality in the feature space as well as employ pooling within a biased frequency region to reduce translational variation and adjust for disturbances and small changes in the feature space [22]. Mukhamadiyev et al. proposed an end-to-end deep neural network-hidden Markov model speech recognition model and a hybrid connectionist temporal classification (CTC)-attention network for the Uzbek language and its dialects. The proposed approach reduces training time and improves speech recognition accuracy by effectively using the CTC objective function in attention model training [23]. Feature extraction and classification using a one-dimensional convolutional neural network (CNN) model, which divides heart sound signals into normal and abnormal directly independent of an electrocardiogram (ECG), was proposed in [24]. They claim that the classification accuracy is higher in the 1D CNN than in the 2D CNN for the heart sound signals used in this study when the convolution kernel is less than 52 because a large convolution kernel can lead to computational complexity and is increasingly time-consuming. In [25], a deep neural network was trained to maximize the posterior accuracy of the state sequences of acoustic models with respect to the speech feature sequences using the TIMIT database (TIMIT Acoustic-Phonetic Continuous Speech Corpus).

## 3. Pre-Processing: Material Overview

For this purpose, the development of rapid algorithms for extracting feature parameters from speech signals and parametric representations, the extraction of helpful and informative samples for processing, and the development of programs for the implementation of spectrograms of selected acoustic segments are indispensable.

Speech recognition systems consist of two main subsystems:

○ The primary processing of speech signals (phonogram);
○ The classification of acoustic symbols using an intelligent algorithm (spectrogram).

The first subsystem generates acoustic symbols as a set of informative acoustic properties of the signals and signal characteristics. The second subsystem converts the speech signal into a parametric form based on the obtained acoustic characteristics. Speech signal processing consists of the following stages:

○ Separation of the boundaries of the speech signal;
○ Digital filtering;
○ Segmentation;
○ Application of a smoothing window;
○ Spectral transformation, and
○ normalization of spectral frequencies.

These steps are used extensively to extract feature parameters from speech signals, and their main features are considered in the following.

### 3.1. Separation of Boundaries

The extraction of only parts of speech from the incoming signal or the determination of the starting and ending moments of a sentence in a noisy environment is an essential task in speech processing. The following properties of the speech signal are used to solve this problem: the short-term energy of the speech signal, the number of points intersecting at zero, the density of the distribution of the values of the silence field within the signal, and the spectral entropy. Traditional speech recognition systems use techniques that are based on the transient and spectral energies of a signal (e.g., voice activity detection) to determine the speech boundaries from incoming speech signals [26,27,28].

The main parameters of the speech signal are the short-term energy of the speech signal and the number of points passing through zero. As a rule, the parameters of a speech signal change rapidly over time; therefore, it is customary to process a speech signal into fragments of 10 to 30 ms in length that do not overlap. The speech signal is stationary within this range, and areas of silence in the speech signal are removed by calculating the transient energy. The algorithm for solving this problem is divided into *N* sequences of 256 short-term energy values of the next speech signal. 

The calculation of the energy of each fragment by dividing the speech signal is appropriate for parallel and distributed computing. Each fragment is independently processed. If processing is applied in an n-core multiprocessor, it will be possible to calculate the energy of n fragments simultaneously. Therefore, it is possible to increase the parallel processing efficiency during this stage [29,30,31,32].

### 3.2. Number of Zero-Crossings

A zero-crossing is a point at which the sign of a mathematical function changes (e.g., from positive to negative), which is represented by an intercept of the axis (zero value) in the graph of the function [33]. The zero-crossing frequency in a signal may be the most straightforward indicator of its spectral properties. For example, the number of points that pass through zero in a speech signal can be determined using the following equation:

(1) $$Z=\frac{1}{2}\overset{N}{\underset{i=1}{\sum }}\left|\mathrm{sign}\left(x_{i}\right)-\mathrm{sign}\left(x_{i-1}\right)\right|$$

where *x*_*i*_ is the current signal value, *x*_*i*−1_ is the previous signal value, and sign(*x*_*i*_) is the sign function.

During the recording process, the 200 ms portion at the beginning of the speech signal is considered the silent portion. The silent values are random variables. The distribution density of the sample value of the silent field is used to separate the speech from the incoming signal. These algorithms are traditional tools for separating speech signals from areas of silence in a phonogram. The implementation of these algorithms is simple and does not require large amounts of computing time or memory. However, these algorithms are less effective under non-stationary noise conditions and in the presence of various sound artifacts. Algorithms based on flexible instantaneous values have also been proposed. However, these algorithms also become unstable when disturbances such as sound artifacts, relatively high noise levels, or insignificant levels of useful signals occur. Therefore, researchers have developed effective algorithms for the stable determination of speech boundaries, even in the presence of non-stationary interactions, based on spectral analysis.

The following are the main requirements for the methods that are used to determine the boundaries of speech under the influence of noise [34]:

- Ensuring a minimum probability of error under the influence of high noise levels,
- the probability of the correct detection of useful speech, even under loud noise conditions;
- High speed in determining the speech boundaries that meet the real-time mode requirements.

**Figure 1:**
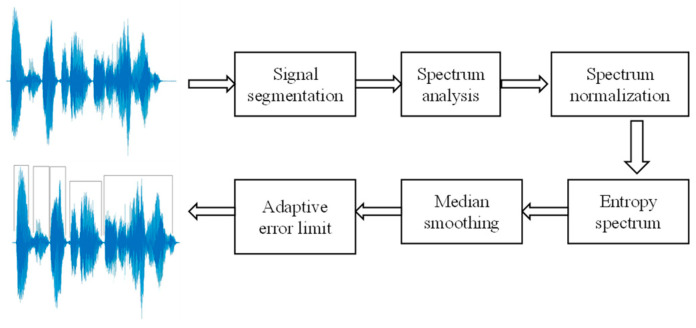
Method of speech separation based on spectral entropy analysis.

The spectral entropy method can also be applied to meet these requirements. This method is based on the calculation of the information entropy of the signal spectrum. The difference in the entropy values for the speech signal and background noise segments is used to determine the speech boundaries. A distinctive feature of this approach is that it is insensitive to changes in the signal amplitude. The process of determining speech boundaries based on spectral entropy consists of six steps, as illustrated in Figure 1.

### 3.3. Digital Filtering

In addition to a typical, useful signal, various types of noise are present. As noise negatively affects the quality of speech recognition systems, dealing with noise is a pressing issue. Two types of digital filters are used to reduce the noise levels in the system: a line filter and an initial filter. A linear filter can be considered a combination of low- and high-frequency filters as it captures all low and high frequencies. Initial filtering is applied to minimize the impact of local disturbances on the characteristic markings that are used for subsequent identification. A speech signal must be passed through a low-pass filter for spectral alignment [35].

### 3.4. Segmentation

Segmentation is the process of dividing a speech signal into discrete, non-overlapping fragments. The signal is usually divided into speech units such as sentences, words, syllables, phonemes, or even smaller phonetic units. The segmentation of recordings that contain the utterances of numerous speakers may consist of attributing pieces of utterances to particular speakers. The term “segment-stations” is sometimes used to refer to a division of the speech signal into frames prior to its parameterization [36,37].

### 3.5. Spectral Transformation

It is necessary to distinguish the main features of speech signals that are used in the later stages of the speech recognition process. The initial features are determined by analyzing the spectral properties of the speech signals. The fast Fourier transform algorithm is commonly used to obtain the spectral frequency of a speech signal [38,39].

### 3.6. Normalization of Spectral Frequencies

The entire computational process in intelligent algorithms is based on moving decimal numbers. Therefore, the parameters of the objects that are classified using neural networks are limited to the range [0.0, 1.0]. The resulting spectrum is normalized between 0 and 1 to apply spectral processing using the neural network. To achieve this, each vector component is divided into its maximum components.

Spectral processing methods enable the use of all data samples that are obtained from the speech signal. Many speech signals have a specific frequency structure and spectral properties. Spectral methods provide high-precision processing of speech signals. The disadvantages of spectral processing include low flexibility in the local characteristics of the signals, a lack of spectral dimensions, and relatively high computational costs.

Fourier transformation, wavelet analysis, and many other algorithms are used extensively in the spectral processing of speech signals. Fourier transformation is used in many fields of science, including speech processing. In speech signal processing, Fourier transformation converts the signal from the time field to the spectral field as a frequency component [40]. 

**Figure 2:**
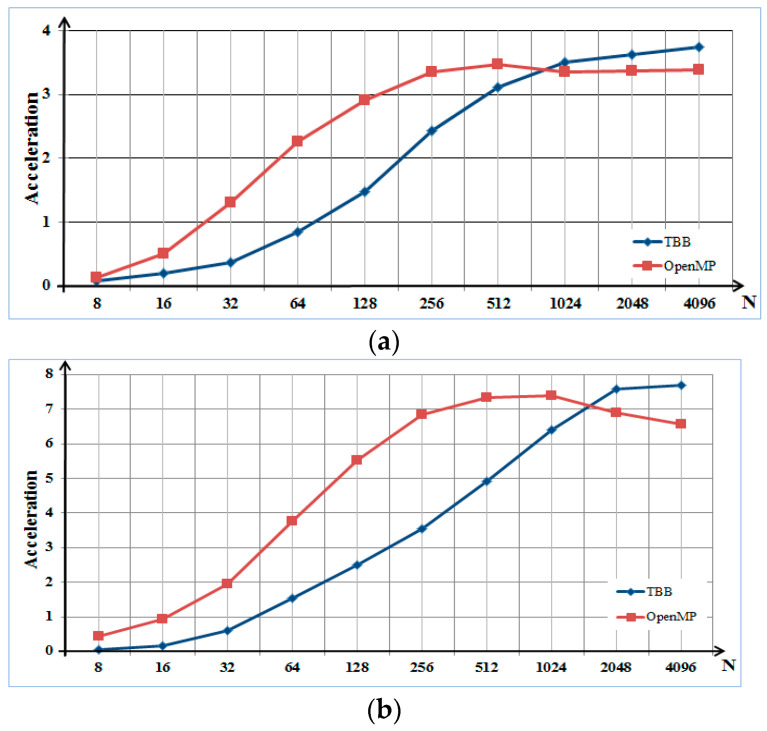
Acceleration results for (**a**) four- and (**b**) eight-core processors.

In previous studies, discrete Fourier transform (DFT), discrete cosine transform (DCT), short-term Fourier transform, and wavelet transform were applied for spectral analysis. These methods were used to transform fragments of a speech signal into the frequency domain and calculate the spectrum. The TBB and OpenMP packages were used to create parallel algorithms for spectral transformations. In these methods, the command divides the signal into frames; for example, N = 16, 32, …, or 4096 for each frame [4]. The size of each created frame is equal to the block size of the cache memory because cache memory is a factor that affects the efficiency of parallel processing. The acceleration results are depicted in Figure 2.

For the software implementation of the parallel processing algorithms, serial and parallel processing were performed on four- and eight-core processors, which were applied using personal computers and servers, as listed in Table 1.

The main feature of the DCT algorithm is periodicity. An advantage of DCT is that it tends to concentrate most of the signal energy on a small number of factors. The following equations provide the elements of the coefficient matrix:

(2) $$L\left(0\right)=\sqrt{\frac{1}{N}}\overset{N-1}{\underset{n=0}{\sum }}x\left(n\right)$$

(3) $$L\left(k\right)=\sqrt{\frac{2}{N}}\overset{N-1}{\underset{n=0}{\sum }}x\left(n\right)\cos\left(\frac{2n-1}{2N}\right)k\pi ,k=1,2,\ldots N-1$$

where *L*(*k*) is the value of the DCT, *k* is an index of coefficients, *x*(*n*) represents data items, and *N* is the frame size.

**Figure 3:**
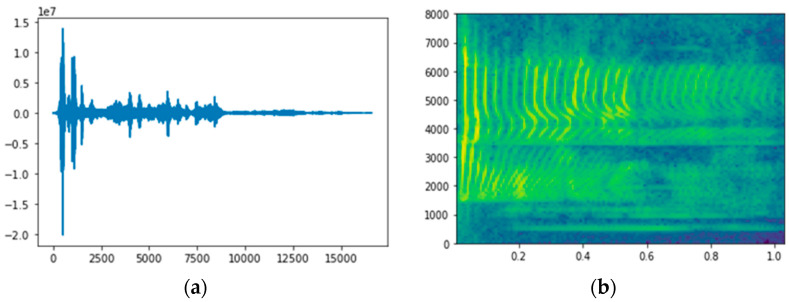
DFT: (**a**) change in frequency range within time domain and (**b**) spectrogram.

Fourier analysis parameters are the basis of the methods that are used to generate feature vectors in most speech recognition systems in the digital processing of speech signals within the spectral domain. Mel-frequency cepstral coefficients (MFCCs) [41] and linear predictive coding (LPC) techniques [42,43] are used in Fourier analysis to generate spectrogram images from speech signals. The spectra that are obtained using Fourier analysis provide concise and precise information regarding a speech signal, as illustrated in Figure 3.

Two-dimensional (2D) signals were used for spectral analysis in the experiments. The 2D spectral analysis characteristics that are used in parallel image-processing algorithms are suitable for parallel processing in multicore processors [38]. In particular, forward and inverse transformation vectors and matrices have binary dimensions, and the compatibility of this multicore processor architecture can effectively increase the computational speed. Therefore, we applied sequential and parallel processing on a computer with different processor characteristics for the hardware implementation of the algorithms that were used for processing 2D signals (Table 2). 

**Figure 4:**
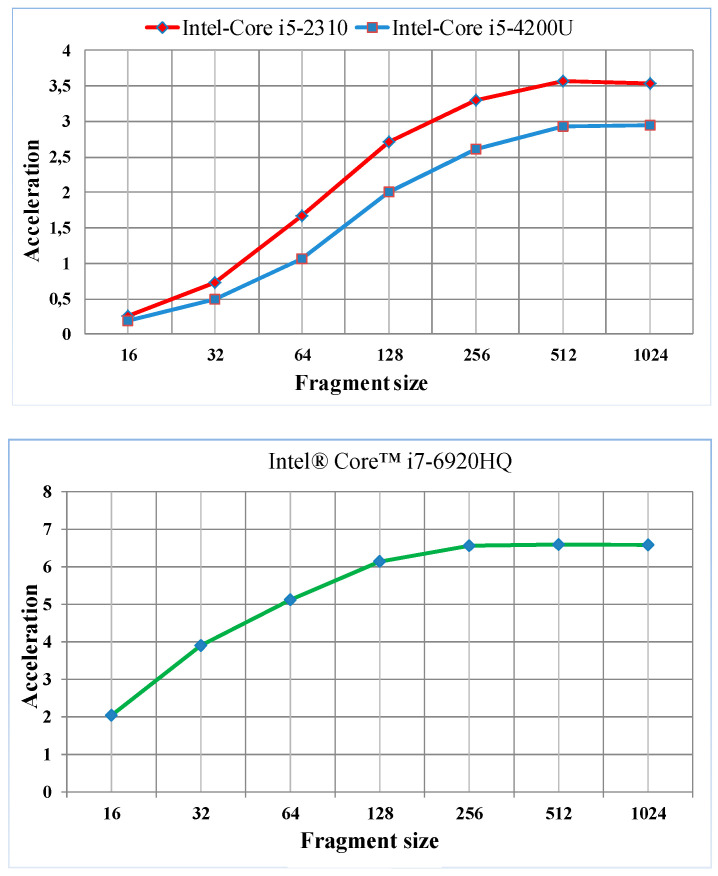
Acceleration results for 2D parallel spectral analysis.

The entire 2D signal was divided into identical fragments of different sizes at each stage [44]. The values of the selected fragments were between pixel resolutions of 16 × 16 and 1024 × 1024. The speed of the parallel DCT algorithm using the OpenMP package compared with that of the sequential algorithm is presented in Figure 4.

The use of a parallel 2D spectral analysis algorithm on multicore processors yields an overclocking result that is close to the number of cores.

### 3.7. Dataset

Dataset collection and generation processes were performed as follows. In this study, the dataset with 120 h of audio was used for the model training. The dataset includes speech audio recordings which consist of sentences with a maximum of 15 words with a total length of approximately 120 h.

In addition, the dataset includes a large amount of different text for use in developing the language model. Over 90,650 utterances, 415,780 words, and 65,810 unique words that were included in the text corpus were collected, resulting in around 120 h of transcribed speech data. We split the dataset into training, validation, and test sets. The dataset statistics are reported in Table 3.

We split the dataset into three folders corresponding to the training, validation, and test sets. Each folder contains audio recordings and transcripts. The audio and corresponding transcription filenames are the same, except that the audio recordings are stored as WAV files, whereas the transcriptions are stored as TXT files using the UTF-8 encoding. All the transcriptions are represented using the Latin alphabet consisting of 29 letters and the apostrophe symbol. To prevent overfitting, we applied data augmentation techniques based on velocity perturbation and spectral enhancement.

## 4. Proposed Method

An important task for programmers when developing speech recognition systems is the creation of an optimal method for the parametric expression of speech signals [45]. This method enables excellent separation of sounds and spoken words while ensuring that speakers are insensitive to pronunciation patterns and changes in the acoustic environment. Most errors in word recognition are caused by a change in the pitch of the signal owing to a shift in the microphone or a difference in the pitch of the pronunciation [46]. Another common cause of errors is random nonlinear deformations of the spectrum shape, which are always present in the speech signal of a speaker [47,48]. Therefore, one of the most important tasks in creating effective speech recognition systems is the selection of a representation that is sufficient for the content of the analyzed signal as well as insensitive to the voices of speakers and various acoustic environments. 

The system that is used for extracting feature parameters typically has the following requirements. The information content, that is, the set of feature parameters, must ensure the reliable identification of recognizable speech elements. Furthermore, the loudness, that is, the maximum compression of the audio signal, and the non-statistical correlation of the parameters must be minimized. Independence from the speaker must also be achieved, that is, the maximal removal of information relating to the characteristics of the speaker from the vector of characters. Finally, homogeneity, which refers to the parameters having the same average variance and the ability to use simple metrics to determine the affinity between character sets, must be provided [49]. However, it is not always possible to satisfy all requirements simultaneously because such requirements are contradictory. The parametric description of the speech elements should be sufficiently detailed to distinguish them reliably and should be as laconic as possible.

In practice, the speech signal that is received from a microphone is digitized at a sampling rate of 8 to 22 kHz. Serial numerical values are divided into speech fragments (frames) with a duration of 10 to 30 ms, which correspond to quasi-stationary speech parts. A vector of features is computed from each frame, which is subsequently used at the acoustic level of speech recognition. At present, a wide range of methods is available for the parametric representation of signals based on autocorrelation analysis, hardware linear filtering, spectral analysis, and LPC. The most common approach for speech parameterization is the spectral analysis of signal fragments and the calculation of their cepstral coefficients.

**Figure 5:**
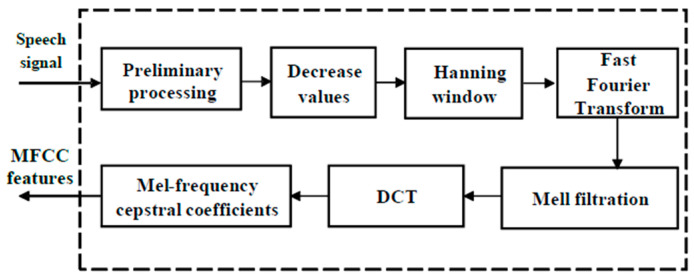
Classical scheme for calculating MFCCs.

MFCCs have been used as informative features for the speech signal [41]. These characteristics are used extensively in speech recognition and are based on two main concepts: the cepstral and Mel scales. The main advantages of the algorithm are its high level of familiarity and ease of speech. The MFCC features are separated from the recorded speech signals. The MFCC algorithm uses the results of the phonogram and spectrum switching algorithms. The classical algorithm that is used to calculate the MFCCs is depicted in Figure 5.

**Figure 6:**
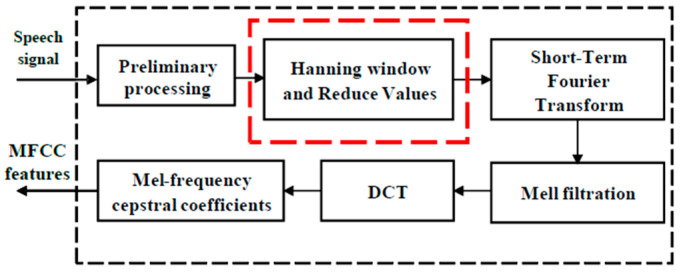
Proposed framework for calculating MFCCs.

This study presents a rapid method for extracting the function parameters from a speech signal. The proposed algorithm for the rapid calculation of the MFCCs is shown in Figure 6.

We consider the execution sequence of the proposed algorithm for the rapid extraction of the function parameters from a speech signal.

### 4.1. Division into Frames

Following preliminary filtering, the speech signal is divided into 16 ms frames. Every frame (except for the first) contains the final 10 ms of the previous frame. This process continues until the end of the signal. In this study, because the sampling rate of the speech signal is 16 kHz, the frame length is N = 256, and the offset length is M = 160. The overlap is 62.5% of the frame length. A coverage of 50% to 75% of the frame length is generally recommended.

### 4.2. Hanning Window and Decreasing Values

A Hanning window size of 1D was used. The Hanning window is also called the raised cosine window. The Hanning window can be thought of as the sum of the frequency spectrum of three rectangular time windows. It can use the side lobes to cancel each other out, eliminating high-frequency interference and energy leakage. Hanning windows are very useful window functions.

A weight box is used to reduce distortion and smooth the individual frames. The floating signal that is examined in this study consists of a dense tone. The magnitude of the flat tone is determined by the magnitude of the pure tone at frequency *f*, which is filtered through the Hanning window.

A critical aspect of this window is that it sets the borders of the frames to zero. In this case, the short energies can be calculated while passing through the window, and they can be transferred from the sequence that is used to calculate the lowest amplitude energies. The aim is to remove low-energy signals from the signal by calculating the signal energy while smoothing the signal from this window. This process requires the following signal energy equation:

(4) $$E_{n}=\overset{N}{\underset{.}{\sum }}x_{i}^{2}$$

where $\;E_{n}$ is the energy of the input signal fragment, and $\;x_{i}$ is the signal value.

**Figure 7:**
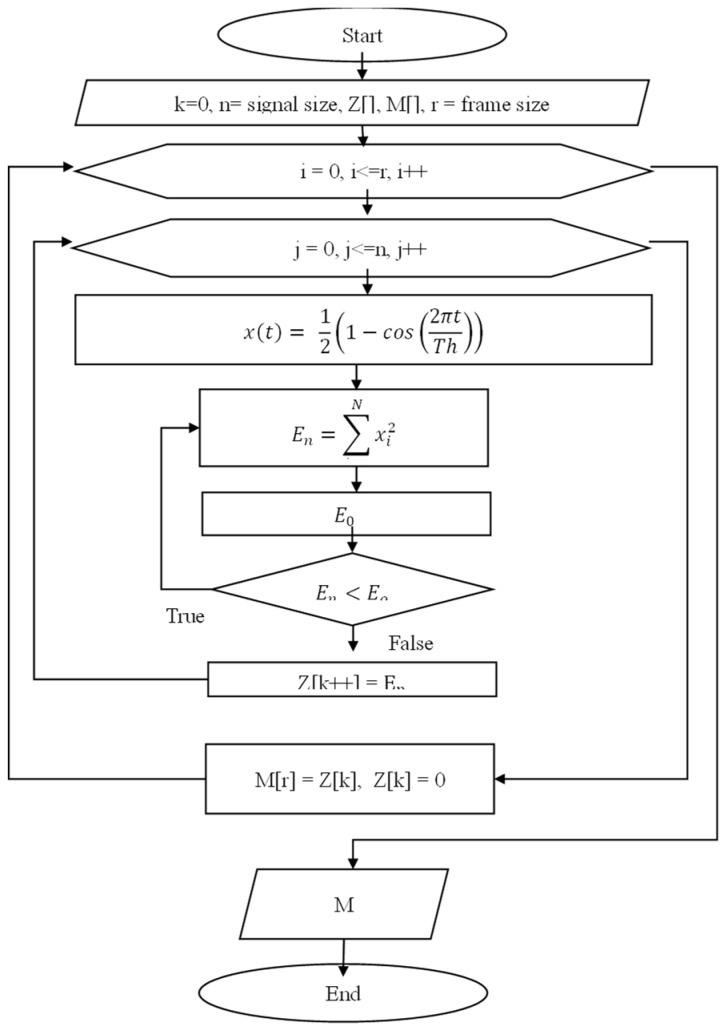
Hanning window algorithm for removing silent parts.

In addition to the window process using (11), the signal is processed in the next step, which significantly reduces the number of values that enter the processor. Figure 7 presents the parallel processing algorithm.

The window size represents a number of samples and a duration. It is the main parameter of the analysis. The window size depends on the fundamental frequency, intensity and changes in the signal.

### 4.3. Short-Time Fourier Transform (STFT) Switches

An intuitive understanding exists of the meaning of a high or low height. *STFT* is a Fourier-related transform that is used to determine the sinusoidal frequency and phase content of local sections of a signal as it changes over time. In practice, the *STFT* computation involves the division of a longer time signal into shorter segments of equal length, followed by a separate calculation of the Fourier transform on each shorter segment. This reveals the Fourier spectrum of each shorter segment.

Discrete-time signals are used in practice. The corresponding time-frequency conversion is a discrete Fourier conversion, which describes the length of signal *X*_*n*_ as representative of the complex value frequency domain of the *N* coefficients. *STFT*, which describes the evolution of the frequency components over time, is one of the most widely used tools for speech analysis and processing [50]. Similar to the spectrum itself, one advantage of *STFT* is that its parameters have physical and intuitive interpretations. *STFT* is typically visualized using the log spectra 20log10 (*X*(*h*, *k*)). Such 2D log spectra can then be viewed using a thermal map known as a spectrogram.

In the third stage of the algorithm, the *STFT* spectral switching procedure is applied to the frames that are passed through the weight window. The *STFT* of the signal is obtained by opening the windows and determining the DFT of each window. In particular, the transformation for the *X_n_* and *W_n_* windows of the input signal is determined as follows:

(5) $$STFT\left\{x_{n}\right\}\left(h,k\right)=X\left(h,k\right)=\overset{N-1}{\underset{n=0}{\sum }}x_{n+h}w_{n}e^{-i2\pi \frac{kn}{N}}$$

where the *k*-index corresponds to the frequency values, and *w_n_* is the window function, which is commonly a Hanning window or Gaussian window that is centered around zero. 

### 4.4. Mel Transform

In the fourth stage, the signal that is transferred to the frequency band is divided into ranges using triangular filters. The filter boundaries are calculated using the chalk frequency. The transition to the chalk frequency field is based on the following equation:

(6) $$B\left(f\right)=1127*\ln\left(1+\frac{f}{700}\right)$$

where *f* is the frequency range.

The reverse switch is determined as follows:

(7) $$B^{-1}\left(b\right)=700{\left(e^{\frac{b}{1127}}-1\right)}_{xx}$$

Consider *NN* as the number of filters (26 filters are generally used) and flows, as high as the frequency range under study. This range is transferred to the Mel scale and divided into *NN* evenly distributed intersecting ranges. The linear frequency-appropriate boundaries are determined within the field, whereas the weighting coefficients that are obtained based on filtration are denoted by *H*. Subsequently, the filters are applied to the square modulus of the coefficients obtained from the Fourier transform. The values that are obtained are logarithmic owing to the following expression:

(8) $$e_{m}=\ln\left(\overset{N}{\underset{k=0}{\sum }}{\left|x_{k}\right|}^{2}H_{m,k}\right),m=0,1,2\ldots N-1$$

The singular value decomposition algorithm is implemented in the final stage of calculating the MFCCs.

## 5. Experimental Results

**Figure 8:**
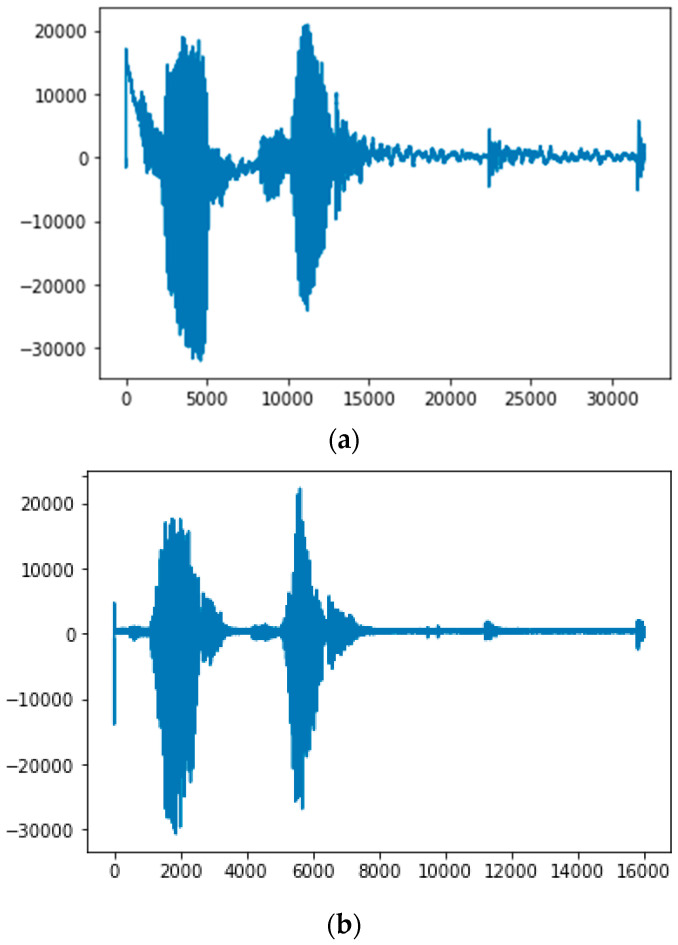
(**a**) Initial incoming signal and (**b**) appearance of signal after applying proposed algorithm.

We implemented and tested the proposed method in Visual Studio 2019 C++ on a PC with a 4.90 GHz CPU, 32 GB of RAM, and two Nvidia GeForce 2080Ti GPUs, as shown in Table 4. The system was tested in different device environments to evaluate the performance of the signal feature extraction method. In the experiments, during the operation of the algorithm (Figure 8), when the sequence of surfaces of the signal was *n* = 15, the number of values was reduced by 40–50%, and the processing time increased 1.2-fold. Consequently, this algorithm exhibited significantly higher efficiency. Moreover, this algorithm enabled the separation and elimination of areas of silence while passing through the Hanning window.

**Figure 9:**
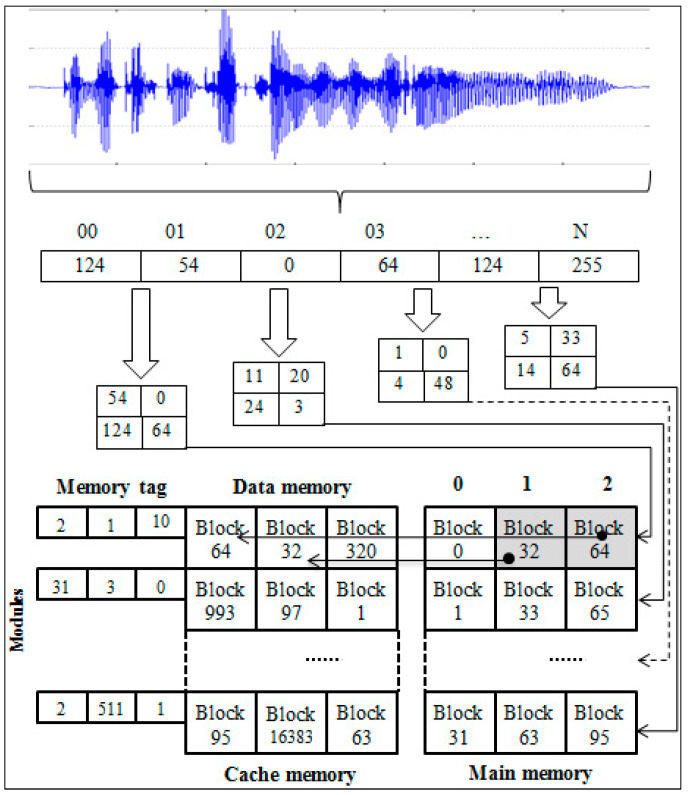
Parallel computing structure using RK3288 processors.

Several possible means are available for avoiding the waste of memory bandwidth. We propose a new solution that increases computing performance by matching the size of the signal frames to a block size of cache memory. This type of optimization can significantly affect the overall parallel processing performance. However, it can be used in digital signal processing by dividing the signal into frames through implementations on multicore processors. However, in practice, the selection is usually performed on a small scale, corresponding to the width of the data bus that connects the cache memory to the main memory and the size of its block. Our method implements the optimal use of these memories in parallel computing. The organization of the cache memory plays an essential role in parallel processing algorithms when dividing data into streams. In particular, the presence of vector-matrix effects in digital signal processing and the size of their streams should be adjusted according to the size of the cache blocks. This can be achieved using the proposed method, as illustrated in Figure 9. 

**Figure 10:**
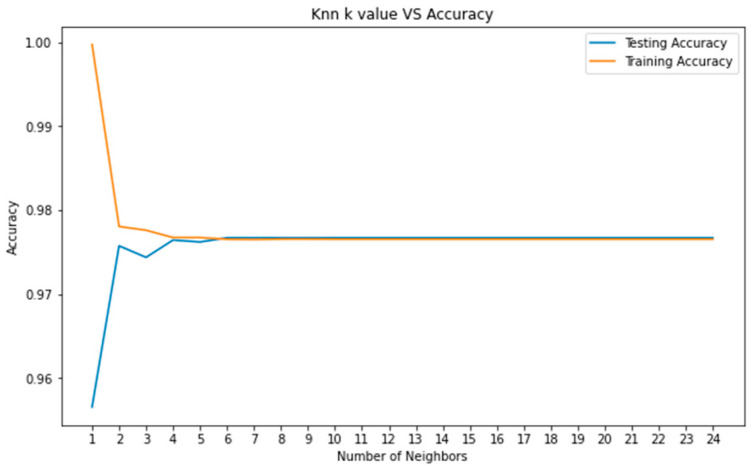
*k* value of KNN algorithm (with feature selection).

In this section, we discuss a quantitative analysis to compare the performance of different systems. We compared our method with well-known speech recognition algorithms based on deep-learning approaches. Evaluation metrics are essential for computing various strategies for speech recognition and assessing the performance of different approaches. Although we used the results of other studies for comparison, we are not certain whether they are true because the source codes and datasets of these methods are not publicly available to verify the actual performance. Figure 10 depicts the result of removing the silent parts during the passage of a speech signal fragment through the Hanning window based on the proposed rapid algorithm. The speed results obtained from the analysis are presented in Table 5.

A range has been determined in order to find the degree of the neighborhood that gives the best accuracy value in the KNN algorithm. The specified range covers 1–25. In Figure 10, the graph of the KNN algorithm with feature selection was applied. When the graph was examined, when the neighborhood value was 1 at the beginning, the training accuracy was much higher than the test accuracy. In the KNN algorithm created using the features selected by correlation, the accuracy of the model was determined to be 99.15% in the training dataset and 97.35% in the test dataset.

The word error rate (WER) or character error rate is typically used to evaluate the accuracy of feature extraction from a speech signal. These are objective matrices that are helpful for a fair comparison of recognition techniques. In our previous studies [51,52,53,54,55,56], we computed metrics such as the F-measure (*FM*), precision, and recall. The *FM* is the weighted average that balances the measurements between the precision and recall rates. The precision is the ratio of the number of correctly predicted positive observations to the total number of predicted positive observations. The recall is the ratio of the number of correctly predicted positive observations to the total number of observations in the actual class, as indicated in (9). The following equations can be used to calculate the average precision and recall rates of feature extraction methods:

(9) $$Precision=\frac{TP}{TP+FP}\;Recall=\frac{TP}{TP+FN}$$

where *TP* denotes the number of true positives, *FP* denotes the number of false positives, and *FN* denotes the number of false negatives. 

The *FM* is calculated using (10), considering both precision and recall.

(10) $$FM=\frac{2\times precision\times recall}{precision+recall}$$

The average *FM*, *recall*, and *precision* of the proposed method was 98.4%. False detection occurred in 1.6% of cases owing to the unwanted noise of signals at the microphone. The range of the model’s accuracy was between 0 and 1, and the metric estimation scores reached their best values at 1. An evaluation of our method and other recently published speech feature extraction methods is presented in Table 6. The same number of features was used for a fair comparison. A total of 325 speech samples from each group were analyzed from subjects with similar feature backgrounds. The effects of different frame lengths according to the number of filter banks in the MFCC and different frame lengths in the order of the LPC were also examined for improved accuracy.

As mentioned previously, the WER is the most common measure for speech recognition performance. It is computed by comparing a reference transcription with the output of the speech recognizer. Based on this comparison, it is possible to calculate the number of errors, which typically belong to three categories: (1) insertions when a word is not present in the reference in the output of the automatic speech recognition (ASR), (2) deletions when a word is missed in the ASR output, and (3) substitutions when a word is confused with another word. The WER can be computed as follows.

(11) $$\mathrm{WER}=\frac{S+D+I}{N}$$

where *S* is the number of substitutions of words that are incorrectly recognized, *D* is the number of deletions, *I* is the number of insertions, and *N* is the number of words in the reference transcription. The main issue in computing this score is the alignment between the two-word sequences. This can be determined through dynamic programming using the Levenshtein distance [67].

**Figure 11:**
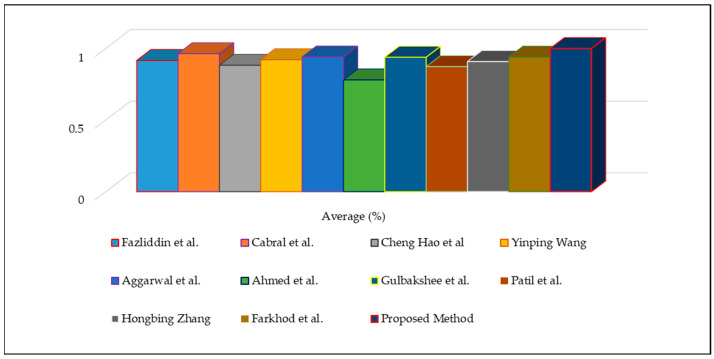
Quantitative results of speech signal feature extraction approaches using vertical graphs.

Based on Table 6, we performed a statistical analysis to indicate the average accuracy of the compared methods using the WER evaluation metric, as illustrated in Figure 11. The improved feature extractor yielded an accuracy of approximately 98.4%, whereas the other approaches yielded accuracies between 78% and 96%. We used the results provided in the relevant papers for comparison; however, the accuracy of these values is not easily verifiable because the source codes and datasets of these methods are not publicly available to confirm their real performance. Nevertheless, in the case of standard scenes, the proposed method was experimentally demonstrated to provide excellent speech feature extraction accuracy by reducing the computational time, even when the speech data are noisy or of low quality.

**Figure 12:**
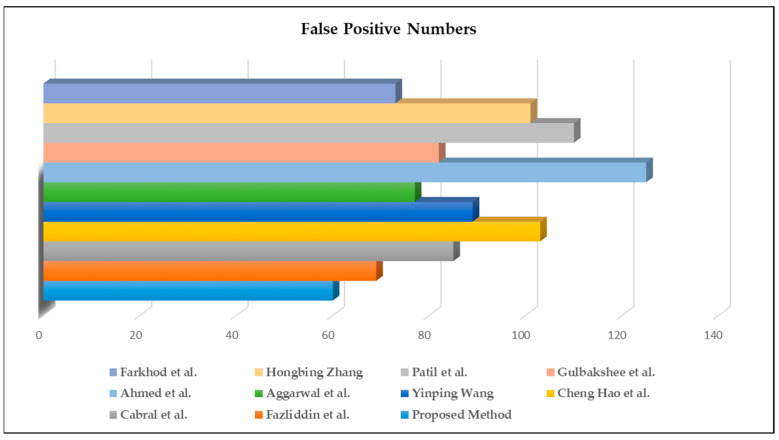
Visible results of false-positive speech signal feature extraction experiments.

Moreover, we evaluated the false-positive results of the selected methods. As can be observed from Figure 12, the proposed approach had the fewest errors. Furthermore, the highly efficient parallel computation method significantly reduced the sound signal feature selection and extraction errors. Overfitting was one of the main issues during the training, and almost all machine learning models suffer from it. We tried to reduce overfitting risk using a feature selection technique that aims instead to rank the importance of the existing features in the dataset and discard less important ones (no new features are created).

Table 7 displays the performance results of the methods that were used in speech recognition environments based on different properties. Our proposed approach does not suffer from unwanted and unnecessary background noise and is not affected by low-quality human voices such as hoarse voices, voices produced with a sore throat, or even sounds from humans with complete voice loss. Our method aims to overcome the problems of inadequate recording equipment, background noise, difficult accents and dialects, and various pitches in a voice. In a normal environment, the best results for accurately detecting and extracting speech feature challenges were obtained using the proposed method with a reduced processing time.

The results of the speech recognition methods were classified as powerful, normal, or weak for the seven categories. The powerful criterion demonstrates that the algorithm can overcome all types of challenges. In contrast, the normal criterion indicates that the algorithm may fail in certain instances because the word boundaries are not defined beforehand. Finally, the weak criterion suggests that the algorithm is unreliable under background noise or vibrations.

## 6. Limitations

It is difficult to conclude that the methods proposed to date do not exhibit any shortcomings. Our proposed method may also result in errors owing to various noise environments. To overcome this problem, we aimed to reduce the number of features in the dataset by creating new features from existing features [69]. Since overfitting was one of the main issues for training different models during the competition, enriching the training data by adding data samples from different resources could be a possible solution for improving the results. Irrespective of the aforementioned problems, the experimental results revealed that our method was very robust and effective for speech feature extraction tasks, with an average accuracy of 98.4% and *FM* of 99.5%.

## 7. Conclusions

A novel high-performance parallel computing approach using a machine learning method has been proposed for speech recognition systems. Acceleration problems in machines with limited computing resources can be solved using distributed systems. The computation speed in signal recognition systems can be increased, and the performance of multicore platforms can be improved by creating and using efficient and rapid algorithms. The results demonstrate that the proposed model reduces the processing time and improves the feature extraction accuracy by 98.4% by effectively using MFCCs. It has been observed that the features with low correlation values extracted by feature selection are also effective in the success of the model. The statistical analysis was performed on the pre-processed data, and meaningful information was produced from the data using the K-nearest neighbors (KNN) machine learning algorithm.

Future studies will focus on improving the accuracy of our method by using deep learning approaches and optimizing the cache memory of multicore processors to detect and extract speech signals without a significant quality loss. Furthermore, we plan to construct a spectral analysis model based on parallel processing with robust analysis performance that will enable the establishment of embedded devices with low computational resources using Taris speech datasets [70] in the 3D CNN and 3D U-Net Environment [71,72,73,74,75].

## Figures and Tables

**Table 1:** Characteristics of Intel processors.

Processor Model	Clock Frequency (GHz)	Cores	Cache Memory Processor L1/L2/L3
Intel Core i5-2310	3.0	4	64 KB/256 KB/6 MB
Intel Core i7-7700	3.60	4 (8)	256 KB/1 MB/ 8 MB
Intel Xeon E5-2609 v4	1.77	8	64 KB/256 KB/ 20 MB
Intel Core i7-11800H	2.30	8 (16)	640 KB/10 MB/24 MB

**Table 2:** Characteristics of applying Intel processors.

Processor Model	Clock Frequency (GHz)	Cores	Cache Memory Processor L1/L2/L3
Intel Core i5-2310	3.0	4	32 KB/256 KB/6.0 MB
Intel Core i5-4200U	2.3	2/2	32 KB/256 KB/3.0 MB
Intel Core i7-6920HQ	3.80	4 (8)	256 KB/1.0 MB/8.0 MB

**Table 3:** The dataset specifications.

Category	Train	Valid	Test	Total
Duration (hours)	109.56	5.04	5.4	120
# Utterances	82.7	3.8	4.08	90.650
# Words	378.0 k	17.4 k	18.7 k	415.0 k
# Unique Words	60.2 k	2.7 k	2.9 k	65.8 k

**Table 4:** The detailed specifications of the experimental setup.

Classification	Environment
Graphic Processing Unit	GeForce RTX 2080 Ti 11 GB (2 are installed)
Central Processing Unit	Intel Core 9 Gen i7-9700k (4.90 GHz)
Random Access Memory	DDR4 16 GB (4 are installed)
Storage	SSD: 512 GB HDD: 2 TB (2 are installed)
Motherboard	ASUS PRIME Z390-A
Operating System	Windows 10
Language	Visual Studio 2019 C++
Local Area Network	Internal port—10/100 Mbps External port—10/100 Mbps
Power	1000 W (+12 V Single Rail)

**Table 5:** Experimental results of proposed method.

	Test	Test 1	Test 2	Test Cycle (50)
Algorithm				
Classical		0.0399	0.0459	5.7758
Our method		0.0407	0.0387	5.2277

**Table 6:** Quantitative accuracy results of speech feature extraction.

Algorithm	Number of Features	Precision	Recall	FM	Average
Fazliddin et al. [57]	325	0.918	0.921	0.914	0.911
Cabral et al. [58]	325	0.975	0.961	0.963	0.963
Cheng Hao et al. [59]	325	0.817	0.945	0.872	0.882
Yinping Wang [60]	325	0.975	0.879	0.917	0.926
Aggarwal et al. [61]	325	0.937	0.942	0.943	0.941
Ahmed et al. [62]	325	0.773	0.787	0.805	0.78.9
Gulbakshee et al. [63]	325	0.921	0.924	0.932	0.938
Patil et al. [64]	325	0.876	0.858	0.879	0.872
Hongbing Zhang [65]	325	0.904	0.893	0.907	0.908
Farkhod et al. [66]	325	0.935	0.938	0.942	0.938
Proposed method	325	0.983	0.987	0.995	0.984

**Table 7:** Review of speech feature detection and extraction performance using various features.

Criteria	Ayvaz et al. [10]	Gogate et al. [68]	Mukhamadiyev et al. [23]	Proposed Method
Robust to Vibration	weak	normal	powerful	powerful
Timing of words	normal	powerful	normal	powerful
Difficult accents and dialects	weak	normal	weak	normal
Speaker identification	normal	normal	weak	powerful
High-dimensional signal space	weak	normal	powerful	powerful
Background noise	powerful	normal	normal	normal
Computation speed	normal	powerful	normal	powerful

## Data Availability

Not applicable.
